# Modelling human visual navigation using multi-view scene reconstruction

**DOI:** 10.1007/s00422-013-0558-2

**Published:** 2013-06-19

**Authors:** Lyndsey C. Pickup, Andrew W. Fitzgibbon, Andrew Glennerster

**Affiliations:** 1 School of Psychology and Clinical Language Sciences, University of Reading, Reading, RG6 6AL UK; 2 Microsoft Research Ltd, 21 Station Road, Cambridge, CB1 2FB UK

**Keywords:** Navigation, 3D perception, Virtual reality, Stereopsis, Motion parallax, Computational modelling

## Abstract

It is often assumed that humans generate a 3D reconstruction of the environment, either in egocentric or world-based coordinates, but the steps involved are unknown. Here, we propose two reconstruction-based models, evaluated using data from two tasks in immersive virtual reality. We model the observer’s prediction of landmark location based on standard photogrammetric methods and then combine location predictions to compute *likelihood maps* of navigation behaviour. In one model, each scene point is treated independently in the reconstruction; in the other, the pertinent variable is the spatial relationship between pairs of points. Participants viewed a simple environment from one location, were transported (virtually) to another part of the scene and were asked to navigate back. Error distributions varied substantially with changes in scene layout; we compared these directly with the likelihood maps to quantify the success of the models. We also measured error distributions when participants manipulated the location of a landmark to match the preceding interval, providing a direct test of the landmark-location stage of the navigation models. Models such as this, which start with scenes and end with a probabilistic prediction of behaviour, are likely to be increasingly useful for understanding 3D vision.

## Introduction

Many studies on 3D representation assume that the parietal cortex generates representations of the scene in an egocentric frame, the hippocampus does so in a world-centred frame, and coordinate transformations account for the passage of information from one frame to another (Andersen et al. 1997; Burgess et al. 1999; Snyder et al. 1998; Mou et al. 2006; Burgess 2006; O’Keefe and Nadel 1978; McNaughton et al. 2006). However, there is little evidence for a well-ordered 3D representation in cortex underlying each of these putative representations. In striate cortex, retinotopic location and disparity tuning provide an anatomical basis for encoding the visual direction and depth of objects relative to the fixation point, but this anatomical regularity is not found in other parts of the cortex representing egocentric and world-centred relationships (DeAngelis and Newsome 1999; Cumming and DeAngelis 2001). And in relation to psychophysical data, there have been few attempts to model and test the processes assumed to underlie the generation of a 3D reconstruction from images, including the distortions that would be predicted to arise from such processing, as we do here.

Of course, 3D reconstruction is not the only way that a scene could be represented (Gillner and Mallot 1998; Glennerster et al. 2001; Warren 2012) and more generally there are many ways to guide actions and navigate within a 3D environment that do not involve scene reconstruction (Gibson 1979; Franz et al. 1998; Möller and Vardy 2006; Stürzl et al. 2008). Together, these come under the category of “view-based” methods of carrying out tasks. By contrast, in the current paper, we focus on reconstruction-based hypotheses for a scene-matching task and the extent to which these are able to account for the pattern of errors displayed by humans faced with the same task. We have examined view-based predictions for the same task in a previous paper (Pickup et al. 2011), and we will present a detailed comparison of the two approaches in a subsequent paper. Here, we focus on the hypothesis that the visual system generates a reconstruction of the scene. If this is what the visual system does when the observer is asked to remember their location in a scene, then we can model the pattern of errors that we would expect observers to make when they try to return to that location.

Using a similar “homing” task, it has often been shown that changing or removing landmarks can bias or disrupt accurate navigation of bees (Cartwright and Collett 1983), ants (Graham and Collett 2002) and humans (Mallot and Gillner 2000; Waller et al. 2001; Foo et al. 2005). By contrast, in our study the structure of the scene remains constant between the reference and the homing interval, but we nevertheless find that the pattern of errors varies systematically with the structure of the scene. It is these systematic variations that are informative about the nature of the representation the visual system uses. In this paper, we attempt to reproduce a similar pattern of errors using two variants of a reconstruction-based algorithm.

### Paper overview

In Sect. 2, we describe the psychophysical experiment measuring navigation errors in a simple homing task in a virtual environment. Sections 3 and 4 describe how a reconstruction algorithm can be used to recover an estimate of the positions of scene landmarks in an egocentric coordinate system and how these estimates, measured in two intervals (“reference” and “homing”), can be combined to form a probabilistic map of navigation end-point locations. We call this the “basic” reconstruction model. Section 5 describes an alternative way of combining the distributions of position estimates that emphasizes the *relative* location of landmarks, so we refer to this as the “shape-based” model.

Section 6 introduces a different type of experiment that allows us to obtain an estimate of the distribution of errors on participants’ representation of *landmark* location (rather than their own location). We compare this to the equivalent distribution that is inferred as part of the modelling of the first experiment. Section 7 compares the ability of the “basic” and “shape-based” models to account for the data, and Sect. 8 discusses our results in the context of models of spatial representation.

## Experiment 1: navigation to a previously viewed location

Participants viewed a simple scene in immersive virtual reality and were then teleported to a different location in the scene from where they had to return to the original location. The paradigm is similar to a previous experiment by Waller et al. (2001). Waller et al. (2001) tried to distinguish different components of the information that participants might be using in a homing task. In their experiment, they identified two candidate locations predicted by two simple heuristics: first, to keep all the landmarks at the same distance from the observer in the two intervals or, second, to keep all the angles between landmarks constant. They found evidence in favour of distance information being important although they admit that the type of virtual environment they used may have contributed to this outcome. Most of the time, only one landmark was visible at a time in their experiment, so angles between landmarks were rarely available visually, forcing participants to rely more heavily on distance information. Unlike Waller et al., we kept the environment the same between the learning and test phases so there was always a correct location to which participants could return. Naturally, this location is the most likely one, as is confirmed by our modelling, but the distribution of navigation errors that participants make around this point and, in particular, the variation in this distribution with the location of the landmarks in the scene, is something we attempt to predict using a reconstruction model. The experiment and data have been presented by Pickup et al. (2011), but are reproduced here for clarity before introducing the modelling.

### Methods

Five participants took part in the experiment, all with normal or corrected-to-normal visual acuity. Participants viewed the virtual scene using an NVIS SX111 head-mounted display with horizontal field of view of 102$^{\circ }$, vertical FOV 64$^{\circ }$ and binocular overlap of 50$^{\circ }$. The location and orientation of the head-mounted display were tracked at 240 Hz using a Vicon MX3/T20S nine camera tracking system that was used to update the binocular visual display (1,280 by 1,024 in each eye) at 60 Hz with a latency of two frames. The calibration procedure that allows the left and right eye’s viewing frustums to be calculated from the 6 degrees of freedom tracking data is described by Gilson and Glennerster (2012). The size of the physical room in which the participants could walk was 3.5 by 3.5 m. The stimuli consisted of three very long poles coloured red, green and blue so that they could be easily distinguished. Other than the poles, the image was black. The poles were designed so that the only information about their 3D layout was the angles subtended at the eye between pairs of poles and the change in these angles with changes in viewpoint (either by the participant walking or from binocular viewing). The poles were always one pixel wide (anti-aliased) for all viewing distances. The poles extended far above and far below the participant, and when the participant looked up or down by 35$^{\circ }$, the image went black. This prevented participants from ever seeing anything close to a “plan view”. The purpose of this minimalist display was to restrict the number of parameters necessary to model the participant’s navigation errors and to allow different types of model to be distinguished.

**Fig. 1:**
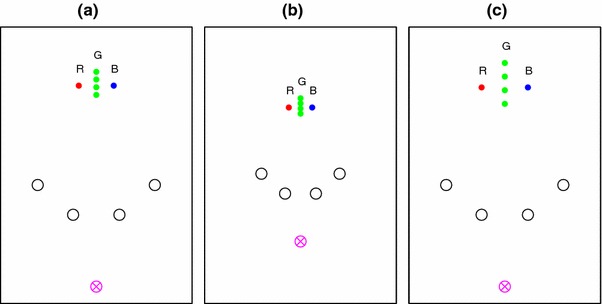
*Plan view of the stimulus layout*. The 48 possible layouts are shown across the *three panels*, each showing four possible positions of the central pole (*green dots*) and four possible centres of the viewing zones (*black circles*), i.e. 16 different configurations per panel. The “goal point”, to which the participant had to return in the second interval, was always within the viewing zone. The *magenta cross* at the base of each plot represents the participant’s position relative to the poles at the start of the second interval. Versions **a** and **b** are identical up to *scale*. Versions **a** and **c** differ in the spatial separation of the set of poles (see text), but are otherwise similar

The layouts of the poles we used are illustrated in Fig. 1. In each case, the red and blue poles and the centre of the viewing zone in the first interval lie on a circle. This means that viewed from each of the four viewing zones shown in a panel of Fig. 1, the angle between the red and blue poles is constant [15$^{\circ }$ for panels (a) and (b), 20$^{\circ }$ for panel (c)].

A trial would start when the participant was within a 20 cm $\times$ 80 cm viewing zone, which was always in the same physical location within the room. It allowed the participant to move laterally to view the stimulus with motion parallax but without the freedom to explore further. The long axis of the viewing zone was always at right angles to a line joining the centre of the viewing zone and the midpoint between the red and blue poles. The participant was instructed to remember their location with respect to the poles. This first “reference” interval ended when the participant pressed a button, and after a 500 ms blank interval, the poles reappeared, but the participant had been transported virtually (i.e. without physically moving) to a new location in the scene, shown by the magenta cross in Fig. 1. The task was to navigate back to the location in the scene at which they pressed the button ending interval one, i.e. the “goal point”. When participants were satisfied that they had reached the goal point, they pressed a button on a hand-held device recording the location of their cyclopean point at that moment and the trial ended. An image then appeared showing a plan view of a schematic head showing their location in the physical room and an outline of the viewing zone to which they had to return to start the next trial.

### Results

**Fig. 2:**
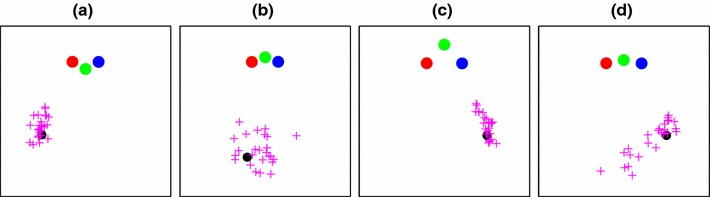
Four examples of navigation-error data, shown as a plan view in a 4 m $\times$ 4 m box. The *magenta pluses* indicate points in the room which subjects reported as being the same as the goal point (*black dot*). The distribution of these points depends on the geometry of the condition: those with a small visual angle between poles tend to have a more “radial” distribution, e.g. conditions **a** and **c**, where the *green* and *blue* poles were seen as being close together when viewed from the goal point. In conditions **b** and **d**, the poles appear more uniformly spaced, and the recorded end-points are more dispersed

The main results of Experiment 1 are shown at the end of the paper in Figs. 11 and 12 where they can be interpreted in relation to the modelling which is described in subsequent sections. However, Fig. 2 illustrates a portion of the data and shows what the main characteristics are that need to be modelled. The black dot shows the goal point to which participants had to return in the homing interval, and the crosses show their end-points. It is clear that the spatial distribution of end-points is affected by the layout of the poles. Figure 2c, d are extreme examples. In (c), the spread of points is mainly along the line joining the goal point and the central pole, while in (d) the pattern in reversed.

In order to gather data that could be plotted in the clear way shown here, i.e. with many trials repeated using exactly the same goal point, we adapted the protocol slightly. Instead of defining the goal point based on the participant’s location in the viewing zone of interval one when they pressed the button, we inserted an “interval 1a” during which the participant saw a static, stereo image of the scene from a fixed viewpoint, and this defined the goal point to which they should try to return in the “homing” interval.

The real data (i.e. the points we analysed and which are shown in Figs. 11) were gathered using only the original two-interval paradigm where the goal position was never exactly the same for different repetitions of a given condition. This does not present any difficulty for modelling, since the goal point was always known, but distributions of errors are more difficult to make out “by eye” when plotted on a unified coordinate frame (see Fig. 12), motivating the use of illustrative “visualization” data as shown in Fig. 2.

## 3D pole position model

If participants are to use a 3D reconstruction in order to recognize a location, there are two steps that must be involved. The first of these, which we consider in this section, is to describe how a reconstruction from *one* location can be generated, including the associated errors. The second, which applies to interval two, is to find a location in the room for which the 3D model of the poles generated in that location best matches the 3D model obtained in the first interval. Errors might then arise if the reconstruction from one location is similar to the reconstruction generated from a different location. In this section, we describe our reconstruction model, and in Sect. 4, we will combine multiple reconstructions from different viewpoints in order to build probabilistic maps representing the likely end-points in homing tasks.

Our starting assumption for a reconstruction-based model is that at each point in the virtual reality space, a participant has access to a reconstruction of the scene which they have built up using stereopsis and motion parallax. Both provide information about the 3D layout of the scene from multiple viewpoints. In the derivation below, we assume that the observer is able to move from side to side, i.e. in a direction perpendicular to the line of sight. This is a good approximation to their movements in the first interval, since the viewing zone was narrow and oriented in this direction, but, of course, we had no control over the participant’s movement in the second interval. We discuss reasons why the model is likely to be robust to a fairly wide range of paths taken by the observer. The following section derives an expression for the expected mean and covariance of the distribution of errors for the three poles for each interval.

### Deriving the distribution over pole positions

**Fig. 3:**
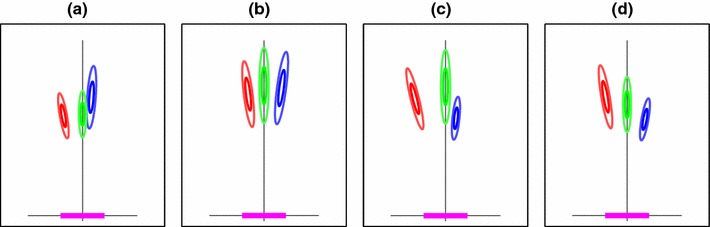
Four examples of the pole-position models, corresponding to the four sets of pole and goal positions in Fig. 2. The *shapes* represent the hypothesized uncertainties over pole locations. Note that the covariances vary according to the distance from the pole to the viewing strip (heavy *magenta line*). In each model, the pole positions are recorded in egocentric coordinates, here represented by the thin $x$- and $y$-axis, so the coordinate frame is independent of the coordinate frame of the room for each condition

The model builds up a reconstruction of the 3-pole scene in an egocentric coordinate frame by assuming there is a set of $N$ cameras all pointing at the central (green) pole and the cameras lie in a strip that extends a distance $\pm w$ along the $x$-axis (where here $w=40$ cm), as shown in Fig. 3. $N$ and $w$ are free parameters in the model. This mirrors the configuration of the “start zone” in interval one which allowed for 80 cm of free motion left and right along an axis perpendicular to the direction in which the green (central) pole lay, while allowing minimal motion in depth (up to $\pm 10$ cm). Participants were asked to step side to side within the start zone. We used the above parameters in the reconstruction model. Since all the information about the 3D room can be captured in its 2D plan view, we consider 1D images of this 2D space, instead of 2D images of the whole 3D virtual environment. The “image noise” on any one of these hypothetical 1D measurements is taken to be Gaussian with a standard deviation of $\sigma$, i.i.d. for each measurement.

The reconstruction we carry out is in an “egocentric” coordinate frame centred on the middle of the start zone, with the line drawn from there to the central pole taken to define the depth axis. This defines the coordinate frame within which the position of each hypothetical camera is specified, as described below. The pole position distributions we obtain as $\left( {{\boldsymbol{M}}}_j, {{\boldsymbol{C}}}_j\right)$ are therefore defined within this egocentric coordinate system.

Assume the poles are ${{\boldsymbol{X}}}_j$ (3-vectors representing 2D points ${{\boldsymbol{x}}}_j$), and $m_{ij}$ is the image of the $j$th point in the $i$th camera. Let the projection matrix for the $i$th camera be ${{\boldsymbol{P}}}_i$; this is a $2\times 3$ matrix with focal length one unit, aligned on the viewing strip facing the central pole. This operates on the 2D homogeneous coordinates of the egocentric coordinate system (i.e. 3-vectors representing a 2D pole location) and transforms them into 1D homogeneous coordinates (2-vectors) representing image coordinates. An excellent introduction to multi-view geometry and working with homogeneous coordinates can be found in Hartley and Zisserman (2004).

For a single imaged point, $m_{ij}$, the likelihood of obtaining this image point, conditioned on the pole position, is

1 $$\begin{aligned} p\left( m_{ij}|{{\boldsymbol{X}}}_j\right) = \frac{1}{\sigma \sqrt{2\pi }} \exp \left\{ -\frac{ \left( m_{ij} -d \left( {{\boldsymbol{P}}}_i {{\boldsymbol{X}}}_j \right) \right) ^2 }{2\sigma ^2 } \right\} , \end{aligned}$$

where $d(.)$ is the de-homogenizing operation, which turns homogeneous 2-vectors representing 1D image points into scalar values. Assuming the noise is independent, the negative log likelihood of multiple imaged points in multiple cameras can therefore be represented as

2 $$\begin{aligned} -\!\log \left\{ p\left( {{\boldsymbol{m}}}|{{\boldsymbol{Y}}}\right) \right\}&= \sum _{i=1}^K \sum _{j=1}^N \frac{1}{2\sigma ^2} \left( m_{ij} -d \left( {{\boldsymbol{P}}}_i {{\boldsymbol{X}}}_j \right) \right) ^2 \nonumber \\&\quad + KN\log \big \{\sigma \sqrt{2\pi }\big \}, \end{aligned}$$

where there are $N$ poles and $K$ cameras, and ${{\boldsymbol{Y}}}$ is the stack of pole locations, i.e. a 6-vector representing the three 2D points.

To obtain a basic estimate of the distribution of the poles given a set of images, we assume there are no interesting priors on ${{\boldsymbol{Y}}}$ and obtain a maximum likelihood estimate, i.e. find values of ${{\boldsymbol{X}}}_j$ for each $j$ so as to minimize the negative log likelihood.

The non-linearities introduced by the projective operation make it difficult to obtain a closed-form solution for the distribution over ${{\boldsymbol{Y}}}$. In order to keep further computations with these distributions tractable, we fit Gaussian approximations to each one, so that each pole has a 2-vector mean, ${{\boldsymbol{M}}}_j$ (its true location), and $2\times 2$ covariance matrix, ${{\boldsymbol{C}}}_j$. The distribution over ${{\boldsymbol{Y}}}$ is simply made up of these three, so there will be a 6-vector mean and a block-diagonal $6 \times 6$ covariance matrix. The three 2D Gaussians are obtained by first considering that the negative log likelihood above can be separated out into three components (one per pole) plus a constant term

3 $$\begin{aligned} -\!\log \left\{ p\left( {{\boldsymbol{m}}}|{{\boldsymbol{Y}}}\right) \right\}&= \nu _1\left( {{\boldsymbol{X}}}_1\right) + \nu _2\left( {{\boldsymbol{X}}}_2\right) + \nu _3\left( {{\boldsymbol{X}}}_3\right) \nonumber \\&\quad + 3K\log \big \{\sigma \sqrt{2\pi }\big \} \end{aligned}$$

where

4 $$\begin{aligned} \nu _j\left( {{\boldsymbol{X}}}_j\right)&= \sum _{i=1}^N \frac{1}{\sigma ^2}\left( m_{ij}-d \left( {{\boldsymbol{P}}}_i{{\boldsymbol{X}}}_j\right) \right) ^2. \end{aligned}$$

Note that the contribution to the negative log likelihood that comes from each pole’s position ${{\boldsymbol{X}}}_j$ is independent of the other poles.

We then take the Taylor expansion of $\nu _j$ by treating it as a function of $x_j$ and $y_j$ (the $x$- and $y$-components of the $j$th pole, ${{\boldsymbol{X}}}_j$) and expanding about the true pole location, ${{\boldsymbol{M}}}_j$. The definition of a Taylor expansion in two variables up to the second-order term is

5 $$\begin{aligned} f\left( x_0+\delta x, y_0 + \delta y\right)&= f(x_0,y_0) + \left[ \delta x, \delta y\right] \left[ \begin{array}{c}\frac{\partial f}{\partial x} \\ \frac{\partial f}{\partial y}\end{array}\right] \nonumber \\&\quad + \left[ \delta x, \delta y\right] \left[ \begin{array}{cc}\frac{\partial ^2 f}{\partial x^2} &{} \frac{\partial ^2 f}{\partial y \partial x} \\ \frac{\partial ^2 f}{\partial x \partial y} &{} \frac{\partial ^2 f}{\partial y^2}\end{array}\right] \left[ \begin{array}{c} \delta x \\ \delta y\end{array}\right] . \nonumber \\ \end{aligned}$$

If the true pole position is ${{\boldsymbol{M}}} = [x_0,y_0,1]^{T}$, then a general point ${{\boldsymbol{X}}}$ can be represented as

6 $$\begin{aligned} {{\boldsymbol{X}}} = [x_0+\delta x,y_0+\delta y,1]^{T}, \end{aligned}$$

and substituting this into (5) gives

7 $$\begin{aligned} f\left( {{\boldsymbol{X}}}\right)&= \nu _j\left( {{\boldsymbol{M}}}_j\right) + 0 + \left( {{\boldsymbol{X}}}-{{\boldsymbol{M}}}_j\right) ^{T} {\boldsymbol{B}}_j \left( {{\boldsymbol{X}}}-{{\boldsymbol{M}}}_j\right) . \end{aligned}$$

The first term of the RHS is a constant with respect to ${{\boldsymbol{X}}}$, and the zero comes from taking the gradient at the true pole position ${{\boldsymbol{M}}}_j$, which should be the maximum of $\nu _j$. ${\boldsymbol{B}}$ is the matrix of second-order partial derivatives of $\nu _j$ evaluated at ${{\boldsymbol{M}}}_j$, and the overall distribution is therefore approximated as a Gaussian with

8 $$\begin{aligned}&\nu _j\left( {{\boldsymbol{X}}}_j\right) \approx \mathcal N \left( {{\boldsymbol{M}}}_{j},{{\boldsymbol{C}}}_j\right) \end{aligned}$$

9 $$\begin{aligned}&{{\boldsymbol{C}}}_j = 2{\boldsymbol{B}}_j^{-1}. \end{aligned}$$

This means that for a pole at ${{\boldsymbol{M}}}_j$, the uncertainty in its location is described by the covariance matrix ${{\boldsymbol{C}}}_j$. The matrix ${\boldsymbol{B}}_j$ from which ${{\boldsymbol{C}}}_j$ is found can be found analytically from (4) by taking the partial second derivatives with respect to ${{\boldsymbol{X}}}_j$ and evaluating it at the pole’s location.

An example of the types of models this gives for the pole position uncertainty is given in Fig. 3. In Sect. 6, we will compare this to pole position uncertainty data from human subjects, but first we will consider how to combine these pole-position-reconstruction models to form likelihood maps predicting navigation errors.

## Combining models to form maps

We now have the foundation of a reconstruction-based model, but still need additional steps in order to explain the homing behaviour of participants. The problem of a human recognizing an exact location in interval two can be viewed as the task of finding a location in the room for which the 3D model of the poles best matches that obtained in the first interval. Navigation errors then arise when the current pole position model is sufficiently similar to the “template” or “goal-point” model generated in interval one.

Using the Gaussian model described in Sect. 3.1, we compute an egocentric pole-position model for every location (putative end-point) in a wide region around the poles. We then compare each model to the one computed at the goal point. End-points for which the model agrees well with the goal-point model should be assigned a higher likelihood in our map than end-points at which the appearance of the poles is less similar. Overall, high likelihoods in this map mean that we expect participants to press the button more often at this location. A map like this is desirable because the probabilities can be compared directly with observed data for any number of configurations and with other similar models (e.g. Pickup et al. 2011).

A likelihood map over the 2D plane is built up one point at a time by considering the distances between two probability distributions: the model is built with a coordinate frame based around the centre of the viewing strip in interval one, and a second model is built using the current point under consideration at the centre of the viewing strip. The $y$-axis is aligned with the green pole and the $x$-axis is perpendicular to this (see Fig. 3). This means that at each point, two egocentric maps of the world are compared. The comparison is made using the probability distributions on the pole positions, as follows.

The distance between probability distributions can be taken in a number of ways, e.g. the KL divergence of one distribution with respect to the other, or the Mahalanobis distance of one set of pole means with respect to the distribution from the goal location. The measure we use is the Bhattacharyya distance between the two maps, because it is symmetric and has a simple Gaussian form, although using measures such as the others, above, makes little difference to the resulting maps. The Bhattacharyya distance between two Gaussians with means ${{\boldsymbol{M}}}_1$ and ${{\boldsymbol{M}}}_2$ and covariances ${{\boldsymbol{C}}}_1$ and ${{\boldsymbol{C}}}_2$ is given by

10 $$\begin{aligned} \mathcal D&= \frac{1}{8}\left( {{\boldsymbol{M}}}_1-{{\boldsymbol{M}}}_2\right) ^{T} {{\boldsymbol{C}}}^{-1}\left( {{\boldsymbol{M}}}_1-{{\boldsymbol{M}}}_2\right) \nonumber \\&\quad +\frac{1}{2}\log \left( \frac{\left| {{\boldsymbol{C}}}\right| }{\sqrt{\left| {{\boldsymbol{C}}}_1\right| \left| {{\boldsymbol{C}}}_2\right| }}\right) \end{aligned}$$

11 $$\begin{aligned} {{\boldsymbol{C}}}&= \frac{{{\boldsymbol{C}}}_1+{{\boldsymbol{C}}}_2}{2}. \end{aligned}$$

The distance between the two distributions is taken to be proportional to the negative log of the likelihood of the observer being at the same location. So, in our task, for some location ${{\boldsymbol{X}}}$, the likelihood of matching the goal point is

12 $$\begin{aligned} \mathcal L \left( {{\boldsymbol{X}}}\right)&= \frac{1}{Z} \exp \left\{ -\lambda \mathcal D \right\} \end{aligned}$$

where $\lambda$ is included as a free parameter determining how quickly the likelihood should decay with the magnitude of the Bhattacharyya distance, $\mathcal D$; it is analogous to a precision (i.e. $1/\text{ variance }$) term in a Gaussian. The normalizing factor $Z$ is the integral of the exponent over the whole of the 2D plane and thus is also a function of $\lambda$ and the other parameters of the reconstruction procedure. This allows us to calculate, for any point ${{\boldsymbol{X}}}$ on the ground plane, the positions of the three poles in camera (egocentric) coordinates as ${{\boldsymbol{M}}}_R, {{\boldsymbol{M}}}_G$ and ${{\boldsymbol{M}}}_B$ with uncertainties over pole positions given by the covariances ${{\boldsymbol{C}}}_R, {{\boldsymbol{C}}}_G$ and ${{\boldsymbol{C}}}_B$.

Taking the set of poles as a single six-dimensional Gaussian distribution with a block-diagonal covariance matrix (i.e. by stacking the three mean vectors and arranging the three $2\times 2$ covariances along the diagonal of a larger $6\times 6$ covariance matrix), we get a single Gaussian representing the three pole locations as seen from a single point. The likelihood $\mathcal L$ for any point ${{\boldsymbol{X}}}$ on the ground plane can then be found using the Bhattacharyya distance between the 6D Gaussian for the view centred on the goal point, and the Gaussian centred on the point ${{\boldsymbol{X}}}$.

### Example maps

**Fig. 4:**
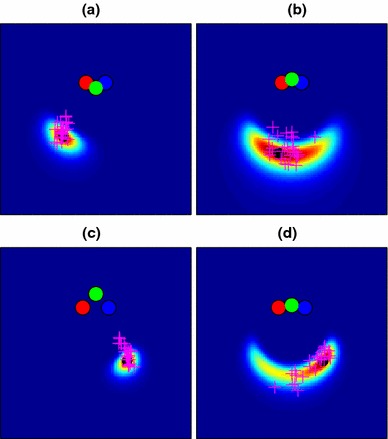
Likelihood maps for the four example conditions of Fig. 2, made using the “basic” map model of Sect. 4. Condition **d**, with particularly high angular uncertainty, is well captured by this model, but the more “radial” distributions of **a** and **c** are poorly explained

Figure 4 illustrates the generation of end-point likelihood maps using the data shown in Fig. 2. The model parameters were set to plausible values: 20 cameras spaced along a line 80 cm in length (i.e. matching the width of the viewing zone), and an “image noise” standard deviation of 0.05 given a focal length of 1m. 480 data points (10 from each of the 48 conditions described in Fig. 1) from a single participant were used to learn an optimal value for the $\lambda$ parameter determining “decay rate” in the Bhattacharyya distance comparison. How well the model fitted these data is described later (Fig. 11), but for illustrative purposes, the fitted model is shown in Fig. 4 in the example conditions from Fig. 2. Our assumption in using the example points for illustration is that both these and the main set of 480 data points are sampled from the same underlying distribution. Note that the elongated “radial” distributions of end-points for cases (a) and (c) are not captured well by this model, though the more loosely clustered points in the other two cases are better explained. In Sect. 5, we will explore a modified version of the 3D model that is better able to account for this pattern of errors.

### Normalization

In order to be able to compare the performance of various maps and to evaluate how well they explain the observed data, it is necessary to turn them into fully normalized probability distributions. We do this by finding the value of $Z$ in Eq. (12), where one distribution is taken to be the reference distribution learnt over the poles in interval one, described by the mean and covariance ${{\boldsymbol{M}}}_0$ and ${{\boldsymbol{C}}}_0$, while the other distribution varies across the plane. Specifically,

13 $$\begin{aligned} Z&= \int \int \exp \!\left\{ \frac{-\lambda }{8}{\boldsymbol{\delta }}^T{\boldsymbol{\varSigma }}^{-1} {\boldsymbol{\delta }} \!+\! \frac{-\lambda }{2}\log \left( \frac{{\boldsymbol{\varSigma }}}{{\boldsymbol{S}}}\right) \right\} \mathrm{d}x \mathrm{d}y, \end{aligned}$$

where

14 $$\begin{aligned} {\boldsymbol{\delta }}&= {{\boldsymbol{M}}}_0({\boldsymbol{\phi }}) - {{\boldsymbol{M}}}(x,y,{\boldsymbol{\phi }}) \end{aligned}$$

15 $$\begin{aligned} {\boldsymbol{\varSigma }}&= \frac{1}{2}\left( {{\boldsymbol{C}}}_0 ({\boldsymbol{\phi }})+{{\boldsymbol{C}}}\left( x,y,{\boldsymbol{\phi }} \right) \right) \end{aligned}$$

16 $$\begin{aligned} {\boldsymbol{S}}&= \sqrt{|{{\boldsymbol{C}}}_0({\boldsymbol{\phi }})||{{\boldsymbol{C}}} \left( x,y,{\boldsymbol{\phi }} \right) |}, \end{aligned}$$

and where ${\boldsymbol{\phi }}$ denotes the set of free parameters in the 3D model of Sect. 3. Thus, the normalizing constant $Z$ depends on the values of the parameters ${\boldsymbol{\phi }}$, and on $\lambda$, which is the free parameter introduced in Sect. 4. It also depends on the reference model $\left( {{\boldsymbol{M}}}_0,{{\boldsymbol{C}}}_0\right)$, so it must be re-calculated for each different configuration of the experiment, in terms of locations of the poles relative to the goal point.

The value of $Z$ is calculated numerically out to a distance of several metres (e.g. 10 m) from the poles in the $x$ and $y$ directions, beyond which point it is assumed to be virtually zero. The integral is performed using four calls the dblquad function from Matlab: since the best match is expected to be at the location where the point ${{\boldsymbol{X}}}$ coincides exactly with the goal point, this point is included explicitly in the integral by splitting the region into four rectangles such that the central corner shared by all four regions is the goal location. This prevents the numerical integral routine missing particularly narrow peaky distributions.

### Assumptions used in the interval-two model

In the computations described above, for every point on the end-point likelihood map (such as Fig. 4) the pole location probabilities are calculated in exactly the same way as they are at the goal location, i.e. using a viewing strip. In the experiments, the participants were free to walk around the virtual reality area in interval two, so no such restriction was made on the space of views of the poles available to them in this interval. In particular, all the views leading up to a candidate end-point could have been integrated together, potentially, into a single pole-position likelihood distribution in the current egocentric coordinates. Assuming, instead, that participants restricted their movement to a narrow viewing strip similar to interval one is clearly an approximation.

**Fig. 5:**
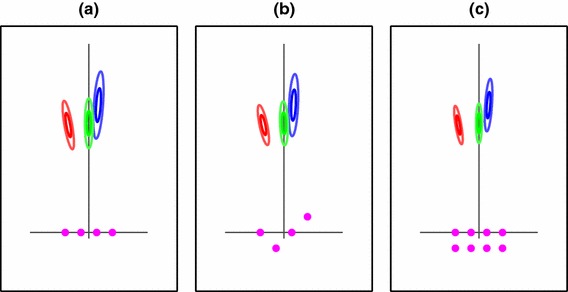
Three egocentric models of pole position, built using variations on the positioning and number of cameras used. In each case, the overall shape of the covariances is the same, though in the case with more cameras, the extent shrinks noticeably. **a** The standard viewing strip, as used in our model; **b** the viewing strip with standard width, but incorporating views from closer or further away; **c** a viewing strip with additional views from behind, as one might expect if a participant has approached the goal location from the starting point

The consequences of this approximation are minimized by three factors. First, anecdotally, the participants did indeed often stand still at an end-point and make the same side-to-side stepping motions as they had been instructed to make in interval one, in order to decide whether they really had reached their goal point although they were not instructed to behave in any particular way in interval two. Second, the starting point for interval two was always farther away from the poles than the goal point was (as shown in Fig. 1). Because the uncertainty on pole location in the reconstruction model is assumed to arise originally from image noise, views from farther away have less influence on the overall distribution for estimating pole position than views closer up, so integrating views along this walking path would have less influence on the distribution than if, for example, participants had walked right up to the poles then backed away to the correct distance. None of the participants did this. Finally, the addition of a small number of extra hypothetical views does not change the pole position model drastically. In Fig. 5, we show the pole distribution for some alternative configurations of the hypothetical cameras. In the case where there are additional views considered—eight instead of four—a general shrinking of the uncertainty is of course seen. If it was the case that all the interval-two covariances were in fact smaller, the maps themselves would not change much because when the models are learnt, this tightening of the distribution is compensated for by a change in the fitted $\lambda$ value.

## Shape-based map

The model described above forms an account of a “basic” photogrammetric reconstruction followed by comparison of two reconstructions from separate intervals. In this section, we explore a variation in the model that incorporates an element of sensitivity to relative positions, since this is known to be important in human vision (e.g.Westheimer 1979).

**Fig. 6:**
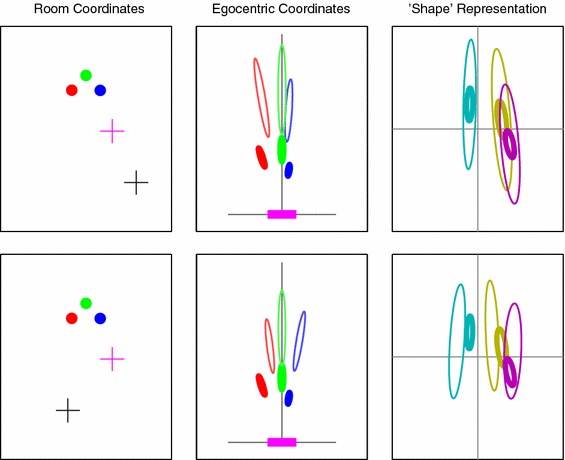
Illustrations of components of the “shape” model. The *left column* shows the room with three poles and two viewpoint (*pluses*). The *magenta* viewpoint, used for reference, is the same on the *top* and *bottom* rows. The *middle column* shows the pole locations, with associated uncertainties (at three standard deviations); the reference view *ellipses* are thick, and the second view of each is drawn in *thinner ellipses*, with *colours* matched to their respective poles. The third column shows the pole-position-difference distributions, where each difference is taken in the egocentric coordinate frame. From *left* to *right*, the three ellipses represent the *blue-to-green* vector, $\left( {{\boldsymbol{M}}}_\beta ,{{\boldsymbol{C}}}_\beta \right)$, the *red-to-green* vector, $\left( {{\boldsymbol{M}}}_\alpha ,{{\boldsymbol{C}}}_\alpha \right)$, and the *red-to-blue* vector, $\left( {{\boldsymbol{M}}}_\gamma ,{{\boldsymbol{C}}}_\gamma \right)$. Again, the *thin ellipses* represent the views from locations marked in *black* in the room-space plot, and the *thick ellipses* mark the views from the reference location. Notice that on the *top row*, where the two viewpoints (*pluses*) and the *green* pole are co-linear, the shape model descriptions align, whereas on the *bottom row* where the viewing angles are different, they do not

We can see that with the “basic” 3D model, defined above, the condition on which its predictions looked least convincing was Fig. 4c, where the green pole appeared much closer to the blue pole than the red one. For all participants, errors tended to show a greater spread in depth for this condition and a smaller spread laterally, whereas the “basic” model does not show this pattern. An alternative model in which the *relative* positions of the poles are the pertinent piece of information remembered from interval one might fare better. We explored a model that computed a distribution over e.g. the red-to-green vector recorded in egocentric coordinates (and the same for the other two possible pole pairs). In this formulation, for a given pole configuration, the red-to-green vector will then be identical for any position of the viewing point along a line from the green pole, since this line defines the orientation of the coordinate frame. Figure 6 illustrates this, showing how two viewing points along one such line give rise to similar means but different covariances in the estimate of relative pole positions, while unrelated viewing positions give rise to quite different estimates of relative pole position.

The algorithm for creating a shape-based description of this type is

1. Find a description of landmarks from the goal point in egocentric coordinates: $\left( {{\boldsymbol{M}}}_R,{{\boldsymbol{C}}}_R\right) , \left( {{\boldsymbol{M}}}_G,{{\boldsymbol{C}}}_G\right)$ and $\left( {{\boldsymbol{M}}}_B,{{\boldsymbol{C}}}_B\right)$ (see Sect. 3).
2. Transform these means into relative-location means by taking pairwise differences (red-to-green, blue-to-green, red-to-blue), i.e.
  17 $$\begin{aligned} {{\boldsymbol{M}}}_\alpha&= {{\boldsymbol{M}}}_G - {{\boldsymbol{M}}}_R,\end{aligned}$$
  18 $$\begin{aligned} {{\boldsymbol{M}}}_\beta&= {{\boldsymbol{M}}}_G - {{\boldsymbol{M}}}_B,\end{aligned}$$
  19 $$\begin{aligned} {{\boldsymbol{M}}}_\gamma&= {{\boldsymbol{M}}}_B - {{\boldsymbol{M}}}_R. \end{aligned}$$
3. Transform the associated covariance for each mean, remembering that the uncertainty *adds*, e.g.:
  20 $$\begin{aligned} {{\boldsymbol{C}}}_\alpha&= {{\boldsymbol{C}}}_G + {{\boldsymbol{C}}}_R. \end{aligned}$$
4. Stack the three 2D Gaussians to give a single 6D shape-based description of the view of the landmarks from this goal location.

Once the description has been found for the goal point, the Bhattacharyya difference between this and the descriptions calculated for other points on the 2D ground plane can be found exactly as for the “basic” model.

**Fig. 7:**
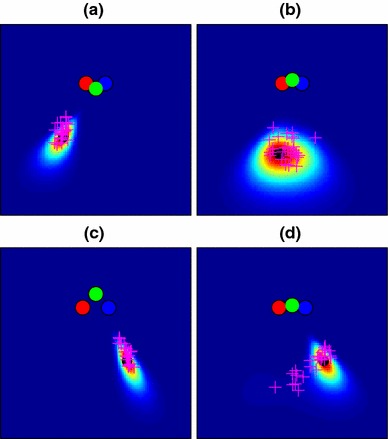
Likelihood maps for the four example conditions of Fig. 2, made using the shape-based map model of Sect. 5. This model assumes that participants remember the layout of the poles relative to one another. A remembered layout from a particular angle therefore matches well to a similar layout seen from slightly farther away, allowing the model to explain the elongated distributions of points in **a** and **c** much better than the basic model of Fig. 4

The shape-based model predictions for the navigation data are shown in Fig. 7 for our four example conditions. This gives the model much better power to explain elongated conditions like those of Fig. 2c, but at a cost, because now the more radially distributed data are less well described than in the basic map, i.e. Fig. 4d shows a better fit than Fig. 7d.

In some ways, the “relative” or “shape-based” model described here is a minor extra step added to the basic model and we are deliberately treating it as such in this paper. This means that the uncertainty that arises, for example, in estimating the location of a pole in the basic model will propagate through and affect the predictions of the shape-based model. That is what makes this a type of reconstruction-based model. However, in another sense, because it is starting to use relative rather than absolute position information, this model is a step down a quite different road, ultimately leading to the abandonment of any type of reconstruction. For example, one could use the relative image locations of pairs of poles as input features to the model and consider independent noise on each of these input measurements. That would be an entirely different, view-based approach, as raised in the Introduction and discussed in a previous paper (Pickup et al. 2011).

The reconstruction model suggests that observers are substantially less sensitive to variations in the depth of a pole than they are to variations in lateral position (Fig. 3). This would, at first sight, seem to run counter to evidence from stereoscopic experiments (e.g. Westheimer and McKee 1979) which suggest the reverse ratio. However, the more relevant data for this experiment are probably those using stimuli with a large disparity pedestal between the reference and the target (McKee et al. 1990) where stereo thresholds can be substantially *poorer* than those for lateral deviations. Here, we designed a method to measure the sensitivity of observers to variations in the position of a given pole in our experiment and so provide a direct empirical test of the distribution of uncertainties over pole position calculated using the reconstruction algorithm, as shown in Fig. 3. The results allowed us to modify the reconstruction stage of the model, as described in the next section.

## Experiment 2: verifying one component of the reconstruction model

In the model presented so far, we set all the parameters in one go; that is, we chose the “decay” parameter, $\lambda$ (Sect. 4) for the model-comparison step at the same time as “internal” parameters, ${\boldsymbol{\phi }}$, for the reconstruction part of the model. In this section, we describe a new experiment that allowed us to separate out the reconstruction parameters and fit them separately, leaving $\lambda$ as a free parameter to be learnt in a subsequent step.

There are two arguments for doing this. First, the reconstruction step itself can be validated in isolation. Second, learning fewer parameters at once reduces the danger of over-fitting and leads to better generalization for the model as a whole. Briefly, the experiment allowed us to probe the underlying shape of the human uncertainty function over pole location. We used the data to fit the standard deviation, $\sigma$, of the noise assumed on the images of the poles, the focal length of the cameras, the number of cameras used, and the width of the strip of cameras (see Sect. 6.3).

### Methods

Participants were shown the three poles from a viewing zone, exactly as in interval one of the trials in Experiment 1, and were asked to remember the layout of the poles. Once participants had memorized the layout, they pressed a button, which led to a 0.5 s blank inter-stimulus interval.

In the second interval, the participant remained in the same location in the virtual scene (unlike Experiment 1) and two of the poles remained in the same place while the third pole was displaced. It was always the same pole that was displaced throughout a whole run although the displacement varied from trial to trial. Participants were told in advance which pole would be displaced. The participant’s task was to move the shifted pole back to the location it had occupied during the first interval. They did this using a hand-held pointing device with which they could “push” or “drag” the pole in two dimensions while pressing a button on the device. Participants indicated that they were satisfied that the location of the pole matched that in the first interval by pressing a different button on the device, advancing them to the next trial. The moving pole always remained vertical, so participants could only manipulate its $(x,y)$ coordinate and, like the other poles, its width in the image was always one pixel (anti-aliased).

### Results

**Fig. 8:**
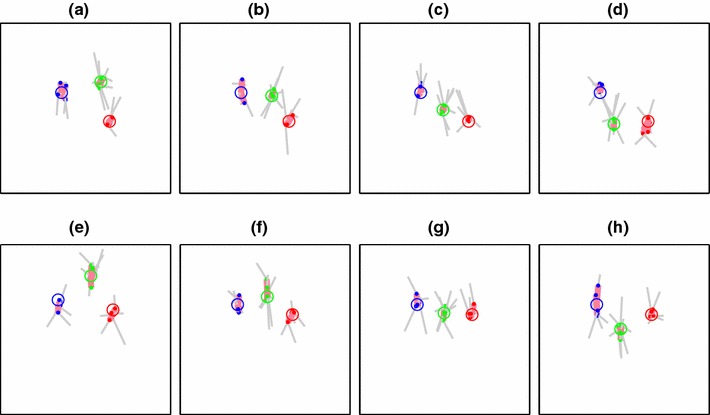
Eight examples from Experiment 2 for one of the participants. The *open circles* represent the correct location of each pole, and the *filled dots* represents the locations at which the participants placed the pole. The *horizontal* and *vertical* axes of the *plots* correspond to the $x$- and $y$-axis of the egocentric coordinate system shown in Fig. 3. *Magenta lines* join the true and estimated pole locations. *Grey lines* link the randomly drawn start location of each pole to the estimated location. *Each plot* shows results from three separate experiments, in which either the *red*, *green* or *blue* pole was movable. *Plots* **a**–**h** show results for eight different configurations of the three poles. Note that in each case, the participant’s own location is not marked because it lies below the bottom of the plots

Examples of the data gathered from Experiment 2 are shown in Fig. 8. This figure includes 80 points from one participant across 16 conditions; in total five subjects completed the task, each providing between 288 and 640 separate pole-position estimates across 32 different conditions. Across the whole dataset, the errors are greater in depth than in a lateral direction. For the green-pole conditions (384 trials in total), the standard deviation projected onto the $y$-axis was 27.2 cm whereas for the $x$-axis it was just 11.7 cm. Similarly, for the 80 points of Fig. 8, which includes all three colours of pole, the $x$-axis standard deviation is 11.9 cm and the $y$-axis standard deviation is 25.2 cm.

In general, this pattern of position uncertainty fits the predictions of the reconstruction model described in Sect. 3 and illustrated in Fig. 3, i.e. elongated in the depth direction. More than this, however, the data allow us to revise the basic and shape models using parameters derived from this uncertainty distribution, as described in the next section.

### Fixing the free parameters, ${\boldsymbol{\phi }}$, using data from Experiment 2

The free parameters, ${\boldsymbol{\phi }}$, in the reconstruction model are: the number of assumed cameras, $N$, image noise standard deviation, $\sigma$, and camera strip half-width, $w$ (see Sect. 3). The model predicts a Gaussian distribution of position errors for each pole, $\left\{ {{\boldsymbol{M}}},{{\boldsymbol{C}}}\right\}$, for which we now have a direct estimate. Hence, we were able learn values for each of these parameters.

In optimizing the data likelihood with respect to these parameters, we found slightly higher likelihoods for the observed data when $w$ was allowed to be a little larger than its veridical value of 0.4 m, which was the half-width of the starting box in the actual navigation experiment. This may be the result of people paying more attention to views at the edges of the viewing space than intermediate views. In our modelling, we limited the width to $\pm$0.4 m in order to reflect the ground truth width of the viewing box.

With the viewing-strip width fixed, $\sigma$ and $N$ were optimized. The latter is a discrete value greater than one, so optimal likelihoods were found for each $N$ as $\sigma$ varied, then the results were compared against each other to find the $(N,\sigma )$ pair maximizing the overall likelihood across the data from Experiment 2. This led to a model using just two cameras, and a noise standard deviation of 0.0128 m when a focal length of 1 m is assumed for the purposes of building up the imaging parameters of the hypothetical cameras. Together with the strip half-width ($w=0.4$ m), these create the reconstruction model which best described human uncertainty in the pole locations in our experiment. Using these same parameters, $\phi$, for this stage of the model and for all participants, we can now return to the second layer of the 3D-based navigation models.

### Revised navigation predictions

**Fig. 9:**
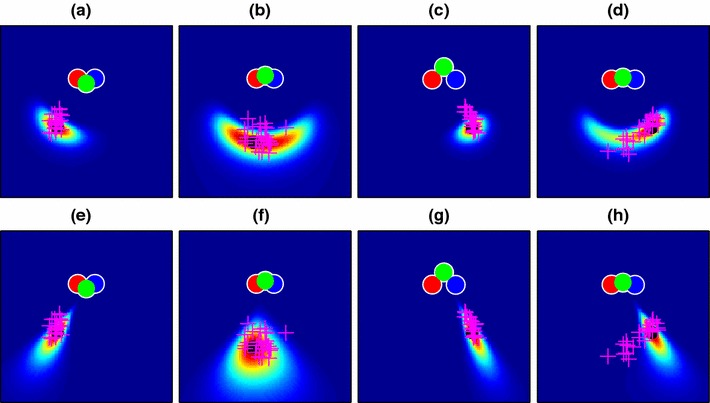
Likelihood maps for end-point location using the optimized 3D pole-position model (with parameters found in Sect. 6) for four example conditions out of the 48 conditions used in the experiment (see Fig. 2 for raw data). **a**–**d** Maps using the basic 3D model; **e**–**h** Maps using the shape-based model

Figure 9 shows the updated predictions for our original navigation data These come from re-running the “basic” and “shape” models described in Sects. 4 and 5 but now using the parameters, $\phi$ obtained in Sect. 6.3 from Experiment 2. Values for the free parameter, $\lambda$ (Sect. 4), are given in Table 1. The shapes of the distributions have changed little compared to the plots of Figs. 4 and 7 and the basic 3D model still fails to provide a good account of conditions like Fig. 9c while the shape-based model provides a better account for conditions like Fig. 9h.

**Table 1:** Weight values, given as $\log _{10}(\lambda )$, for the five participants (P1–P5) on each of the two types of reconstruction-based model

Type	P1	P2	P3	P4	P5
Basic	$-$0.10	$-$0.22	$-$0.31	$-$0.17	$-$0.03
Shape	0.94	0.46	0.39	0.49	0.80

These, along with the parameter values ${\boldsymbol{\phi }}$ obtained in Sect. 6.3 completely specify each of the models

**Fig. 10:**
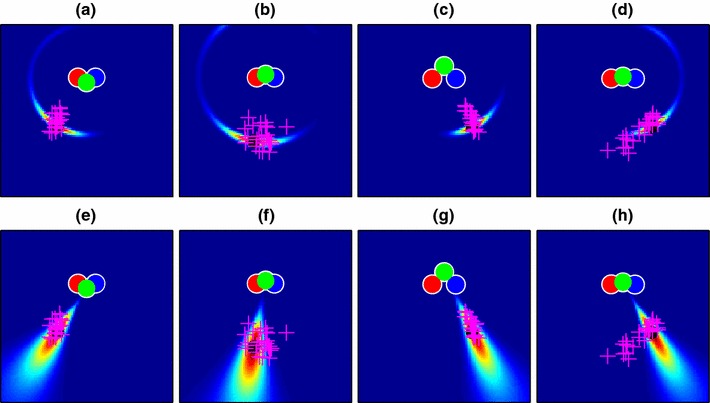
Likelihood maps for end-point location using an *implausible* pole-position model in which the covariance ellipses were rotated through $90^{\circ }$. These provide an unconvincing account of the navigation experiment data compared to Fig. 9

It is not inevitable that the agreement between the two approaches should be so close (i.e. with and without incorporating parameters derived from the data from Experiment 2). For example, if the covariance matrices for the pole positions are rotated by $90^{\circ }$, so that each one describes a data distribution that is elongated in the lateral direction and narrowed in the depth direction, the consequences are quite different, as Fig. 10 illustrates. The prediction of navigation performance is much worse in this case for both the “basic” and the “shape-based” models. This suggests that the data on sensitivity to pole position from Experiment 2 is at least compatible with the navigation data we have observed.

## “Basic” and “shape” reconstruction models compared

**Fig. 11:**
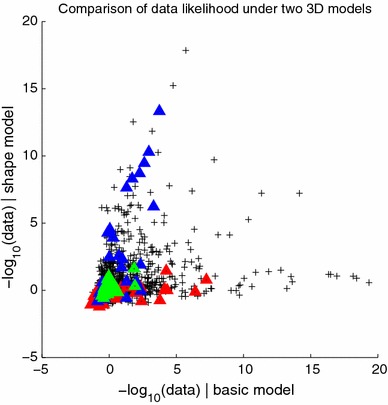
*Scatter plot* comparing the negative log likelihood (a measure of *error*) of each data point under the 3D-basic and 3D-shape models. Each point represents one trial; data are plotted together for 5 subjects across 48 different conditions. Three particular conditions are highlighted using *colours*; these are drawn in the navigation-room space in Fig. 12. In the *red* case, behaviour follows the shape model; in the *blue* case, the basic model. The *green* points belong to a condition on which participants tended to perform well and was explained equally well by each model

We have discussed the visual comparison of the two models provided in Fig. 9, i.e. the “basic” and “shape” models from Sects. 4 and 5, but in this section, we compare the likelihoods of the whole data set under the two models. We do this by computing a likelihood map for the particular pole locations and goal point in any trial and then normalizing it to produce a probability distribution on the 2D plane as described in Sect. 4.2. The probability of the observed end-point for that particular trial under the model can then be read off this map, and compared to the probability of that same data point under the competing model. We carried out this procedure for all 1,776 separate human trials in the navigation dataset (5 participants, each providing between 48 and 480 data points). The overall likelihood of the data under a given model is taken to be the product of all these probabilities, plotted in Fig. 11 as a sum of negative log likelihoods, where low numbers indicate that the data are well explained by the model.

**Fig. 12:**
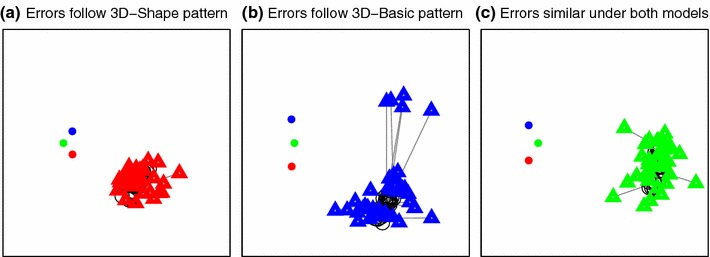
Three example conditions from the navigation experiment illustrated in Fig. 11 using corresponding *colours*. Each end-point is linked by a *black line* to its corresponding goal point (*black circle*); these goals are not exactly coincident because each participant was able to select a goal from anywhere within the viewing box of interval one. The three *coloured dots* to the left of each box show the locations of the poles, and each box is 4 m $\times$ 4 m in size. **a** The *red* condition is well explained with a radial distribution, and so favours the shape model; **b** the *blue* condition shows a high uncertainty laterally and so favours the basic 3D model with its crescent-like distributions; **c** participants performed consistently and well on this condition, and end-points lay close to the means of both models without much spread, so both models performed well

Figure 11 shows the two models compared in this way. Three of the 48 conditions are highlighted using coloured symbols: in the red case, the data are better explained by the shape model than the basic one, but the converse is true in the blue case. The data in each of these three coloured conditions come from different participants and different trials: so, clearly condition is a crucial factor. The red condition is similar to that illustrated in Figs. 2c and 7c, i.e. one in which two poles were close to being aligned at the goal location and so the data had a tendency to be elongated in this direction. The shape model does a much better job of accounting for this pattern of navigation errors, as can be seen in Fig. 7c and confirmed by the red triangles in Fig. 11. Conversely, the blue triangles in Fig. 11 correspond to a condition that is more like that shown in Figs. 2d and 7d where the data form more of a crescent shape and the “basic” model does a better job of explaining this pattern. Figure 12 shows the 37 data points corresponding to each of these three conditions, but for practical reasons, these are harder to interpret than Figs. 2 and 7. This is because the goal point could be selected anywhere in the 20 cm $\times$ 80 cm starting zone, which was not the case for the example conditions shown in Figs. 2 and 7 (see Sect. 2.1). Note that this smearing only affects the illustrations in Fig. 12, and it does not affect the modelling in any way, since each of the 1,776 trials’ probability maps was calculated separately.

What is clear from Fig. 11 is that while *both* reconstruction-based models provide an explanation for a good deal of the variation observed in the human navigation error dataset, neither model is able to outperform the other consistently, and overall they have a tendency to complement one another.

## Discussion

We have demonstrated the extent to which a reconstruction algorithm can account for participants’ performance in a simple navigation task. Any algorithm that is to predict human behaviour successfully in this case must vary its output according to changes in the visual scene and make explicit the way that noise at various stages in the reconstruction process will affect the predicted spatial distribution of errors in the task. We are not aware of algorithms that fulfil these criteria other than those based on the principles of photogrammetry, as we have used here.

Many papers have assumed that the brain generates a 3D reconstruction of the scene without providing a model of the process underlying its construction in the way that we have done here (Luneburg 1950; Blank 1958; Indow 1995; Tolman 1948; Mou et al. 2006; Burgess 2006; Maguire et al. 1999; Gallistel 1989). While often being quite mathematical in their description, these models are nonetheless descriptions of empirical results fitted *post-hoc* rather than describing a reconstruction process and the noise associated with its different stages. For example, Foley (1991) presents a description of distortions in perceived distance and direction based on psychophysical experiments. However, he provides only a minimal hypothesis about the processes that might underlie these distortions. In particular, he suggests that the compression of visual space may be explained by vergence adaptation occurring over many seconds or minutes in his experiments. By compression of visual space he means that “effective binocular parallax”, a value derived from psychophysical judgements, changes over a small range relative to actual binocular parallax (vergence angle). This hypothesis turns out to be contradicted by more recent data: visual space “compression” measured using a related paradigm has been shown to be very similar for long and short periods of fixation, e.g. 2 s periods of fixation interspersed with large changes in vergence so that vergence adaptation could not occur (Glennerster et al. 1996). A more important criticism, however, is that Foley’s hypothesis about the cause of a compression in visual space relies on changes in vergence to different targets. It is mainly an account that explains the distance estimate of fixated targets rather than being designed to explain distortions across a whole scene at once (without vergence changes). If it is true that information is passed from V1 to parietal cortex to hippocampus and that these representations underlie our perception of space, then the modelling of such transformations should refer to more than a single point at the fovea. In that sense, there is a large gap between descriptions of visual space such as Foley’s and current physiological hypotheses about spatial presentation.

The distortions of space that these models describe (Luneburg 1950; Blank 1958; Indow 1995; Foley 1991) do not predict any shift in the peak of the distribution of errors in our task: it remains the case that the most likely location for participants to choose in interval two would be the correct one because the same distortion would apply in both intervals. Others have discussed whether the notion of a distorted visual space remains tenable in the face of increasing psychophysical evidence against the hypothesis (Glennerster et al. 1996; Koenderink et al. 2002; Smeets et al. 2002; Cuijpers et al. 2003; Svarverud et al. 2010). Independent of that debate, the important point here is that for our task, no distortions of the type described by Luneburg and others would be expected.

Navigation often involves proprioception and vestibular cues in addition to vision (Foo et al. 2005; Campos et al. 2010; Tcheang 2010), but in our experiments, these cues on their own were of no value in carrying out the task. The reconstruction model we have applied does assume some nonvisual information is available, but this is for the purpose of fixing the scale of the visual reconstruction, for example from vergence or proprioception. These provide information about the length of the baseline (distance between the optic centres of a pair of cameras), but otherwise proprioception does not contribute to the process of comparing the stimuli in interval one and two. Any model that tried to integrate proprioceptive information in this matching process would need to be quite complicated, involving a subtraction of two coordinates from visual reconstructions generated at the start of the first and second intervals to get a “homing vector” across the two intervals, and a conversion of this visual vector into proprioceptive coordinates. It is not easy to see how a component derived in this way would add to the explanatory power of the model.

Instead, our model relies on matching of representations generated from visual data. In the end, a full description of human navigation will have to account for multiple sources of sensory information and show how these are integrated. This process will almost certainly incorporate a mechanism for weighting different cues according to their reliability (Landy et al. 1995; Ernst and Banks 2002; Svarverud et al. 2010; Butler et al. 2010) but this does not necessarily mean that the optimal coordinate frame in which to carry out such integration is necessarily a 3D one, as we have discussed elsewhere (Svarverud et al. 2010). Indeed, in relation to the data we have presented here, some of the conditions were best explained by a “shape” model which concentrates on the 3D relationship between pairs of features. This approach no longer uses a full reconstruction of the scene using a single coordinate frame and could be regarded as one step towards abandoning 3D frames altogether.

As we raised in the Introduction, reconstruction models are not the only approach to explaining 3D representation and performance in our scene-matching task. In a subsequent paper, we will compare directly the performance of a reconstruction algorithm with that of a quite different, view-based approach.
